# A 1-minute blood test detects decreased immune function and increased clinical risk in COVID-19 patients

**DOI:** 10.1038/s41598-021-02863-2

**Published:** 2021-12-06

**Authors:** Chirajyoti Deb, Allan N. Salinas, Tianyu Zheng, Aurea Middleton, Katelyn Kern, Daleen Penoyer, Rahul Borsadia, Charles Hunley, Bassam Abomoelak, Vijay Mehta, Laura Irastorza, Devendra I. Mehta, Qun Huo

**Affiliations:** 1 Translational Research and Specialty Diagnostic Laboratory, Arnold Palmer Hospital for Children, Orlando Health, 110 Bonnie Loch Court, Orlando, FL 32806 USA; 2 Nano Discovery Inc., 1060 Woodcock Road Suite 131, Orlando, FL 32803 USA; 3 Center for Nursing Research, Orlando Health, 1414 Kuhl Ave, Orlando, FL 32806 USA; 4 Center for Digestive Health and Nutrition, Arnold Palmer Hospital for Children, Orlando Health, 60 Gore St., Orlando, FL 32806 USA; 5 Internal Medicine Group, Orlando Health, 1414 Kuhl Ave, Orlando, FL 32806 USA; 6 Critical Care Medicine, Orlando Health, 1414 Kuhl Ave, Orlando, FL 32806 USA; 7 Department of Chemistry and NanoScience Technology Center, University of Central Florida, 12424 Research Parkway Suite 400, Orlando, FL 32826 USA

**Keywords:** Biological techniques, Biotechnology, Immunology, Biomarkers, Diseases, Chemistry, Nanoscience and technology

## Abstract

Upon infection with SARS-CoV-2, the virus that causes COVID-19, most people will develop no or mild symptoms. However, a small percentage of the population will become severely ill, and some will succumb to death. The clinical severity of COVID-19 has a close connection to the dysregulation of the patient’s immune functions. We previously developed a simple, nanoparticle-enabled blood test that can determine the humoral immune status in animals. In this study, we applied this new test to analyze the immune function in relation to disease severity in COVID-19 patients. From the testing of 153 COVID-19 patient samples and 142 negative controls, we detected a drastic decrease of humoral immunity in COVID-19 patients who developed moderate to severe symptoms, but not in patients with no or mild symptoms. The new test may be potentially used to monitor the immunity change and predict the clinical risk of patients with COVID-19.

## Introduction

Coronavirus disease 2019 (COVID-19), a viral infectious disease that is caused by Severe Acute Respiratory Distress Syndrome Virus-2 (SARS-CoV-2) virus infection, has become a global pandemic following an initial outbreak in the People’s Republic of China at the end of 2019 and beginning of 2020. Until October 2021, 242 million people around the world have been infected with the virus and 4.9 million people have died of COVID-19^1,2^. Upon infection, while most people will show no or mild symptoms, a significant portion of the population will develop moderate to severe symptoms that require hospitalization, aggressive treatments such as mechanical ventilation, intensive care, and some eventually succumb to death^3–5^. The mortality rate based on the number of confirmed positive cases varies from on average 2–3% to as high as ~ 10% in some countries^1^.

Extensive studies have found that the immune response in COVID-19 patients is rather complicated, varies significantly from person to person, and patients’ immune response dynamics have a direct association with their clinical outcome^6–14^. Of particular interest, patients who developed severe symptoms or died of COVID-19 have reduced number of T cells and their immune responses are skewed towards type 2, antibody-mediated immunity^15–21^. These patients are more likely to experience cytokine storm that can lead to uncontrolled inflammation of the body, tissue damage, and eventually organ failure. In contrast, asymptomatic patients or patients with mild symptoms appear to have stronger type 1, cell-mediated immunity than patients with more severe symptoms^22^. In the fight against infectious pathogens, especially intracellular pathogens such as viruses, type 1 immune response is the frontline defense against the pathogen from the immune system. If type 1 immune response fails to stop the infection, type 2 immune response is initiated, clearing the virus by producing virus-specific antibodies^23^. These antibodies include binding antibodies and neutralizing antibodies. While binding antibodies can only bind to virus or virus antigens, neutralizing antibodies bind with the virus in a specific way that render the virus particle no longer infectious or pathogenic^24^. The level of binding antibodies is determined by using immunoassays such as ELISA and quantitated as antibody titer. The level of neutralizing antibodies is analyzed by evaluating the viability of live virus using techniques such as plaque reduction assay. Higher anti-virus antibody titers were found in patients with more severe symptoms compared to those with only mild symptoms^17,18^. On the other hand, neutralizing antibodies with strong potency in the COVID-19 patients is a predictor of survival^25,26^.

While type 1 immunity is dominated with cell-mediated immune responses and type 2 immunity leads primarily to antibody-mediated immune responses, both types are inter-connected through the humoral immune system, particularly, the immunoglobin G (IgG) subclasses. In humans, IgG1 and IgG3 are associated with type 1 immune response, and IgG4 is linked to type 2 immune response^27,28^. In bovine, IgG2 is associated with type 1 and IgG1 is associated with type 2 immunity^29^. In murine models, IgG2a is a specific indicator of type 1 immunity and mouse IgG1 is a marker of type 2 immunity^30^. Under typical, healthy conditions, there is a balance between type 1 and type 2 immunity-associated IgG subclasses. Upon infection or other changes of physiological conditions such as pregnancy or parturition, such balance may be disturbed^29,31^. The ratio of IgG subclasses in blood serum or plasma is often used as a marker to evaluate the relative balance of type 1 and type 2 immune responses in humans and animals^32–34^.

**Figure 1:**
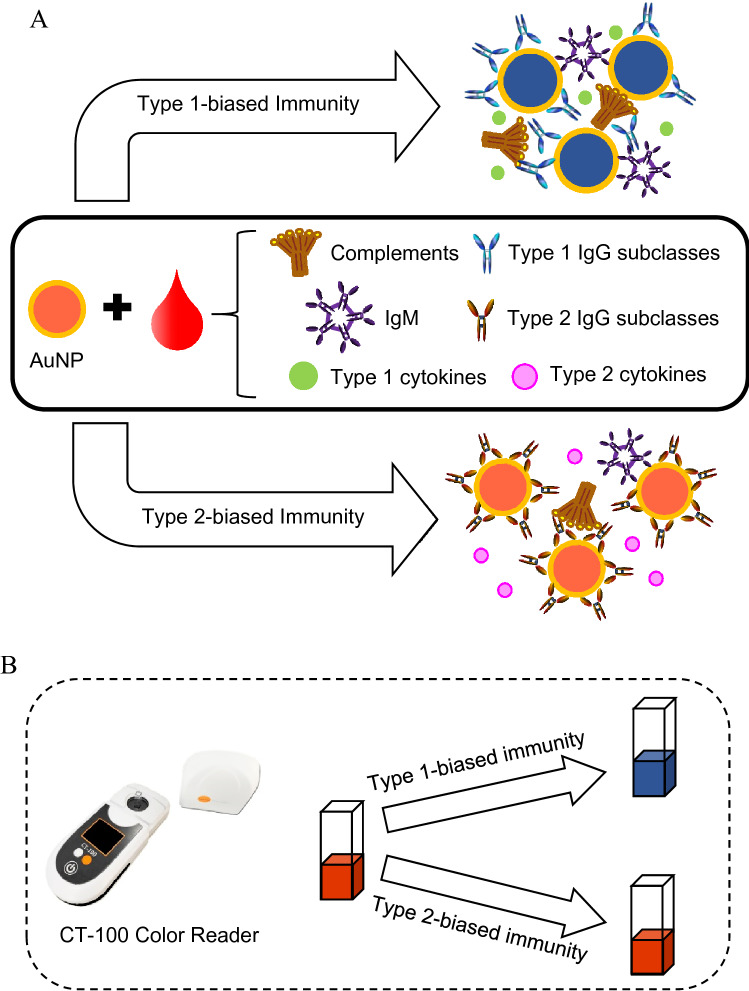
Illustration of the principle of D2Dx immunity test. A gold nanoparticle (AuNP) is used to probe the humoral immune status in a blood plasma or serum sample. The immune interaction between the AuNP and the blood proteins is detected by monitoring the color change of the AuNPs. The color change of the assay solution is measured using a CT-100 colorimeter reader device and the absorbance change over a reaction time of 30 s is expressed as the test score of the D2Dx immunity test.

We previously developed a rapid blood test, D2Dx immunity test, that can detect and monitor the humoral immune status and responses in laboratory and farm animals^35–39^. The test uses a gold nanoparticle (AuNP) as a pseudo virus pathogen to probe the humoral immunity in a blood plasma or serum sample. The AuNP is designed to react with type 1 and type 2 immunity-associated IgG subclasses non-specifically, but differently. Upon interaction with type 1 immunity-associated IgG subclasses, such as human IgG1, IgG3, bovine IgG2 and murine IgG2a, the AuNPs will form large aggregates, leading to a strong color change of the AuNP solution (Fig. 1). This color change is detected using a handheld colorimeter as shown in Fig. 1. The AuNP probe can also interact with type 2-associated IgG subclass, i.e., IgG4 for human, IgG1 for bovine, and IgG1 for murine. However, these IgG subclasses will not lead to AuNP aggregate formation, hence, no color change of the assay solution.

**Figure 2:**
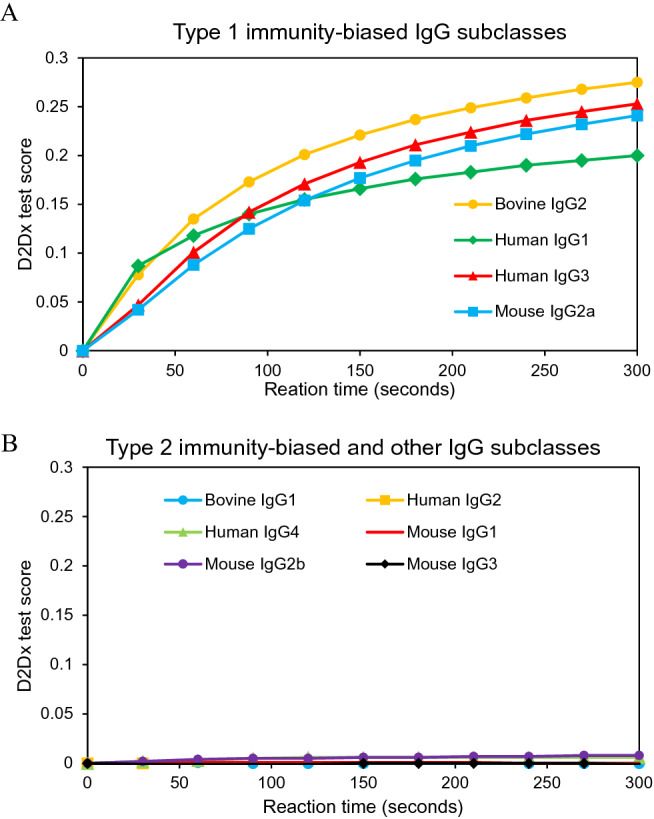
(**A**) Kinetic interaction of AuNP with IgG subclasses indicative of type 1 immunity, including bovine IgG2, human IgG1, human IgG3, and mouse IgG2a. (**B**) Kinetic interaction of AuNP with type 2 immunity-related and other IgG subclasses, including bovine IgG1, human IgG2, human IgG4, mouse IgG1, mouse IgG2b, and mouse IgG3. Kinetic curves shown here are representative of multiple measurements. Graph (**A**) and (**B**) are presented at the same scale for direct comparison.

To illustrate the principle of this test, we present here a kinetic study of the AuNP interaction with different IgG subclasses. Figure 2A is the reaction kinetics between the AuNP probe with IgG subclasses indicative of type 1 immunity, i.e., human IgG1 and IgG3, bovine IgG2, and mouse IgG2a, while Fig. 2B is the reaction kinetics between the AuNP probe and type 2 indicating subclasses, namely, bovine IgG1, human IgG4, mouse IgG1. The association of human IgG2, mouse IgG2b and mouse IgG3 in type 1 versus type 2 immunity is not as clearly understood as other IgG subclasses, but the results are presented here as well. As clearly demonstrated from the study, all type 1-related IgG subclasses, regardless if it is from human, bovine or murine sources, caused dramatic color change of the assay solution; while all IgG subclasses associated with type 2 immunity, led to almost no color change of the assay solution.

In a blood plasma or serum sample, different IgG subclasses will compete to interact with the AuNP probe, and the measured color change of the assay solution is a quantitative indication of the relative balance of type 1 and type 2 immunity. A high D2Dx test score is indicative of a stronger type 1 immunity and weaker type 2 immunity; while a low test score correlates to a weaker type 1 immunity and stronger type 2 immunity. It should be noted here that although the AuNP probe is designed to primarily react with the type 1 and type 2 immunity-associated IgG subclasses differently, other molecules and biochemicals, especially complement proteins are also involved in the interaction with the AuNP probe, as specifically proven in our published study^35^.

Previously, we have used this new test primarily to study and monitor the humoral immune status and type 1/type 2 immunity balance change in laboratory animal models and agricultural farm animals^35–38^. Our studies show that the test can detect active immune response in animals upon virus infections, immune function development in neonatal animals, immune status change in dairy cows associated with pregnancy and parturition. Based on these animal studies, we hypothesize that the D2Dx immunity test may be able to detect the immune status change in COVID-19 patients. Furthermore, if the clinical severity of the COVID-19 patients is associated with the relative balance between the type 1 versus type 2 immunity, the test may be able to detect this difference and the result may be used to predict the clinical risk of the patients.

## Results and discussion

**Figure 3:**
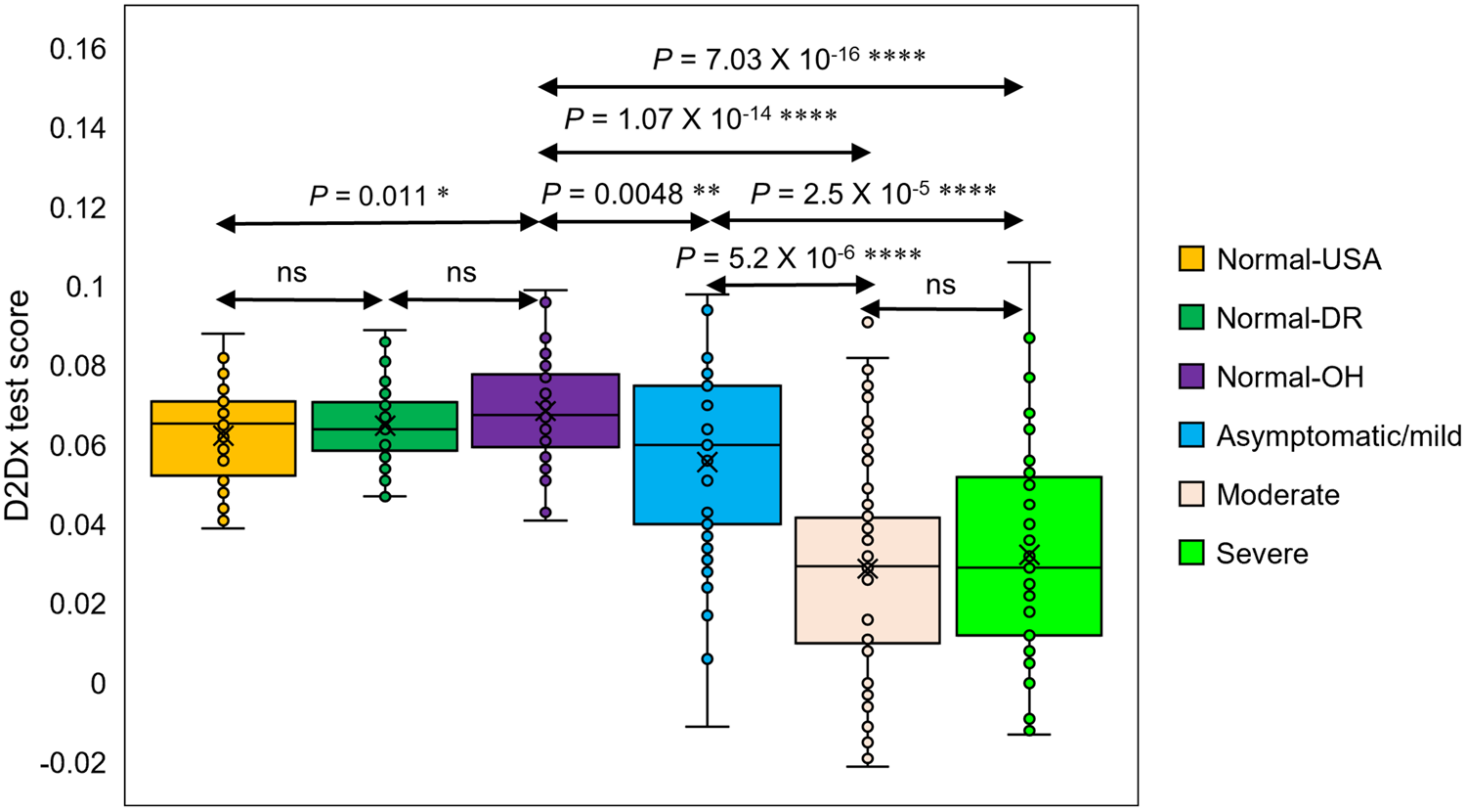
D2Dx immunity test response among various study cohorts. P values between different group pairs were calculated using student t test.

In this study, we tested 142 negative control samples and 153 COVID-19 positive samples (Table 1). Figure 3 is the D2Dx immunity test scores of the six study cohorts. P values calculated from student t-test were listed in the plot for different cohort pairs. Our first statistical analysis was focused on the comparison of the negative control cohort (normal-OH) vs the three COVID-19 patient cohorts, since these samples were collected at the same period from April to August 2020 from the same clinical site, Orlando Health. The average test score of the normal-OH, asymptomatic/mild, moderate and severe cohort is 0.069, 0.056, 0.029 and 0.032, respectively. Statistically significant difference was observed in the following cohort pairs: normal-OH vs asymptomatic/mild (P value 0.0048); normal-OH vs moderate (P value 1.1E−14); normal-OH vs severe (P value 7E−16), asymptomatic/mild vs moderate (P value 5.25E−06), and asymptomatic/mild vs severe (P value 2.47E−05). The difference between moderate and severe cohort is not significant (P value > 0.05). The combined test scores of the moderate and severe cohort are significantly lower than the asymptomatic/mild cohort (P value less than 0.0001).

**Table 1:** Clinical status, collection dates and sample size of different study cohorts.

Study cohort	COVID-19 status	Collection date	Location	Sample size
Normal-USA^a^	Negative	December 2018	USA	42
Normal-DR^a^	Negative	December 2018	Dominican Republic	38
Normal-OH^b^	Negative	April–August 2020	Orlando, FL, USA	62
Asymptomatic/mild	Positive	April–August 2020	Orlando, FL, USA	38
Moderate	Positive	April–August 2020	Orlando, FL, USA	54
Severe	Positive	April–August 2020	Orlando, FL, USA	61

^a^Prepandemic.

^b^OH, Orlando Health.

We also compared the differences between the three negative control cohorts, normal-OH, normal-USA, and normal-DR. There is no difference between the normal-USA and normal-DR cohort (P value 0.296). Although we observed a slight difference between the normal-OH cohort and the normal-USA or normal-DR cohort (P value 0.01), the average test scores of the three cohorts are very close, in between 0.063 and 0.069. Compared to the difference we observed from the negative control versus the COVID-19 positive samples, this difference is rather insignificant. Furthermore, the samples in the normal-USA and normal-DR cohort have been stored for almost two years before testing. It is most likely that the biological activity of these two cohort samples has changed slightly, leading to a very slightly lower average score in these two cohorts (average score 0.062 and 0.064 for normal-USA and normal-DR cohort) than the more recently collected normal-OH cohort samples (average score 0.069). The comparison of the three negative control cohorts confirms that the D2D_X_ test is a highly robust test, and the difference we observed from the normal control samples and COVID-19 positive samples are indeed caused by the patients’ active disease status and different immune responses in the patients.

**Figure 4:**
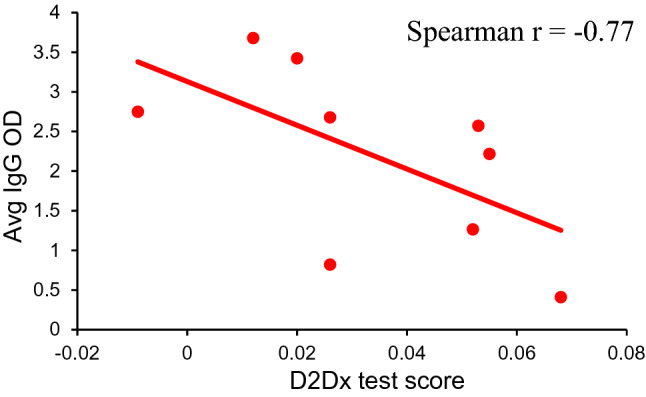
Correlation between D2Dx test scores and anti-SARS-CoV-2 IgG antibody OD values. The IgG value is expressed as the average OD determined by ELISA.

As initially hypothesized, we believe the difference observed from COVID-19 patients with different degrees of clinical severity can be attributed to their difference in type 1 versus type 2 immunity. Patients who developed no or mild symptoms upon infection must have had stronger type 1 immunity and had the capability to maintain this strong type 1 immune response. In D2Dx immunity test, these patients presented relatively high scores. Patients who develop moderate to severe symptoms are those who did not or could not mount a sufficient type 1 immune response, leading to an invoked type 2 response to help control the infection. In this study, we analyzed the anti-SARS-CoV-2 IgG antibody level in 9 randomly selected patient samples. We found a strong negative correlation between the D2Dx immunity test score and the anti-virus IgG titer (Fig. 4), with a Spearman correlation coefficient of − 0.77. This finding, in agreement with many published studies by others^15–22^, provided strong evidence to support our hypothesis that patients that progressed into more severe clinical symptoms developed a strong type 2, antibody-mediated immunity, hence, showed lower scores in the D2Dx immunity test. We also analyzed the anti-SARS-CoV-2 IgM antibody level in 8 out of the 9 samples selected for IgG antibody titer analysis (one sample was not available by the time IgM titer analysis was conducted). We found a moderate positive correlation between the D2Dx immunity test scores and the anti-virus IgM antibody titer, with a Spearman correlation coefficient of 0.53 (data not shown). This observation suggests that the production of IgM antibody at early stage of infection is protective and patients who can produce stronger IgM antibody response may have lower clinical risk^40,41^.

**Figure 5:**
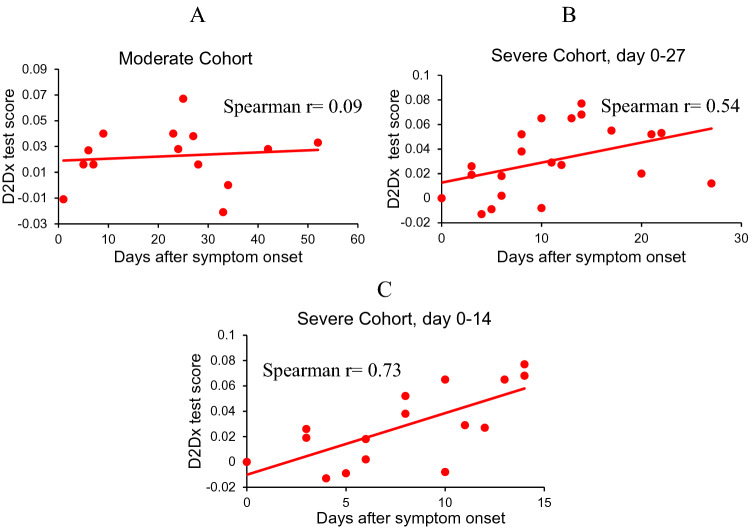
Spearman rank-order correlation of the D2Dx immunity test score of COVID-19 patients in moderate and severe cohort with the days from symptom onset to blood collection. (**A**) Correlation in the moderate cohort (N = 14). (**B**) Correlation in the severe cohort with days from the symptom onset to blood draw in the range of 0–27 days (N = 21). (**C**) Correlation in the severe cohort with days from the symptom onset to blood draw in the range of 0–14 days (N = 16).

Immune response to viral infection is a highly dynamic process. We analyzed the correlation of the immunity test scores of COVID-19 patients in the moderate and severe cohort with their days from symptom onset and blood collection. This analysis is presented in three graphs: Fig. 5A is the correlation analysis of the moderate cohort; Fig. 5B is the correlation of the severe cohort by including 21 samples covering symptom onset days from 0 to 27 days; and Fig. 5C is the correlation of the severe cohort including only 16 samples covering symptom onset days from 0 to 14 days. With the moderate cohort, we found no correlation between the symptom onset days and the immunity test scores (correlation coefficient 0.09). With the severe cohort, when all 21 samples are considered, we found a moderate positive correlation (correlation coefficient 0.54) between the immunity test scores and the symptom onset days. When we limit the days from symptom onset to blood collection to 14 days (2 weeks) in the severe cohort, a strong positive correlation (correlation coefficient 0.73) was found between the immunity test scores and the symptom onset days. Due to limited data, we have in this analysis, the correlation results should be treated with caution. However, the preliminary data indicates that there appears to be a significant dynamic change in the immune response in COVID-19 patients who develop more severe symptoms, while this change is not seen in patients with moderate symptoms.

In summary, we reported here an extremely simple and rapid test that can detect the immune status change in COVID-19 patients and the differences between COVID-19 patients with milder symptoms versus those with more severe symptoms and poor clinical outcome. Although this is a preliminary study, many interesting results were observed and the potential of the test as a point-of-care clinical test for COVID-19 patient management was demonstrated. The test involves a simple one-step process, and the result is obtained in less than 1 min. Although we used blood plasma samples collected through full blood draw in the current study, the test can use blood samples obtained from finger prick, since only a few µL of plasma sample is needed to perform the test. More extensive clinical studies should be conducted to further validate the test. In this study, we did not evaluate the potential influences of clinical interventions, comorbidities, or other demographic factors including age, sex, race or ethnicities of the patients on the test results. Our main objective was to evaluate the applicability of D2Dx test to differentiate three groups of subjects with mild/asymptomatic, moderate, severe disease symptoms after SARS-CoV-2 infection. A small percentage of the patients, especially patients in the severe clinical cohorts received COVID-19 related treatments such as Tocilizumab and/or hydroxychloroquine prior to or at the time of the blood sample collection. These confounding factors may or may not impact the test results and warrant further investigations.

In addition to predicting and monitoring the clinical outcome of COVID-19 patients, the novel D2Dx technology we report here may provide a valuable new tool to accelerate the design and development of new vaccines for COVID-19 and other viral infectious diseases. The balance of Th1/Th2 immune response to a vaccination is an important factor to be considered in the design of the vaccine. A vaccine that primarily elicit a Th2 immune response may prevent the initiation of a Th1 immune response, and sometime may exacerbate the disease following infection^42^. A recently completed study by Swanson et al. shows that the Oxford-AstraZeneca COVID-19 vaccine, AZD1222, induced strong spike-specific CD4 + Th1 and CD8 + T-cell responses in vaccinated individuals^43^. A study by Sahin et al. on Pfizer BNT162b2 vaccine to COVID-19 also revealed a strong type 1 immune response^44^. These two studies suggest an effective COVID-19 vaccine should primarily elicit Th1-biased immune response. With its simplicity, low cost, and rapid results, D2Dx immunity test may be used to quickly assess the Th1 and Th2 response profile generated by vaccination and monitor such responses kinetically over time. Such data can provide further guidance in the design strategies to improve the efficacy of future new vaccines.

## Methods

### Human blood sample sources and collections

All methods were carried out in accordance with relevant guidelines and regulations. The blood samples collected in BD Vacutainer Plus Blood Collection Tubes containing K2EDTA were centrifuged at 2000*g* for separation into cells and plasma as supernatant. The blood plasma samples were obtained from two different sources. Plasma samples of deidentified healthy controls (n = 80) were previously obtained and archived from Boca Biolistics (Boca Raton, Florida) in December 2018 for a different study. Among the 80 samples, 42 were collected in the United States and 38 samples were collected in Dominican Republic. All donors were healthy donors without any reported or known infectious diseases when the samples were collected. The samples were aliquoted and stored at − 80 °C upon receipt until used for this study. Prior to the use for D2Dx assay, the samples were thawed for overnight at 4 °C, and then left to equilibrate at room temperature for 2 h before testing. The samples were tested without any dilution or other treatment.

We collected blood samples from COVID-19 patients and healthy volunteer donors at Orlando Health. The separated plasma supernatants were aliquoted and stored in freezer (− 30 °C). Of which, 62 were from healthy donors and 153 were from COVID-19 positive cases. The study (OH IRB # 20.095.06) was approved by Orlando Health IRB#2. Informed consent was obtained from each patient. Informed consent statement from healthy individual donors was obtained as well. COVID-19 patients were treated at various hospitals within the Orlando Health hospital system as in-patients or out-patients. The IRB also approved to use remnant blood plasma or serum samples from patients and volunteers from a previous serology validation study. The clinical statuses of the study patients were obtained from the patient’s medical record, or by self-reporting by volunteer donors.

### Patient cohorts and demographic information

In this study, we tested 142 negative control samples and 153 COVID-19 positive samples. Table 1 summarizes the clinical status and sample size of each study cohorts. We included three negative control groups. Previously, we collected 80 blood plasma samples from healthy donors in December 2018, about 1 year before the COVID-19 outbreak. The samples were collected at two geographic locations. A total of 42 samples were from the United States (Normal-USA cohort) and 38 samples were from Dominican Republic (Normal-DR cohort). A third cohort of 62 samples were collected at Orlando Health from healthy volunteer donors (Normal-OH cohort) from April to August 2020, in the same time period when we collected COVID-19 positive samples. These volunteer donors were tested negative in anti-SARS-CoV-2 IgG and IgM serology test, and never reported any clinical symptoms associated with COVID-19.

The 153 COVID-19 positive samples were collected between April to August 2020 in Orlando Health (Orlando, Florida). In this study, we used the World Health Organization (WHO) Eight Category Ordinal Scale for Clinical Improvement^45^, as used in the seminal Remdesivir trial^46^, to rank the clinical severity of the patients and group the patients into different cohorts. The uninfected controls are assigned with a 0 score. Patients who were tested positive but exhibited no or only mild symptoms were assigned with a score of 1 (ambulatory with no limitation of activities) or 2 (ambulatory with limitation of activities). These patients were either not hospitalized or were hospitalized for unrelated conditions and found to be positive. From score 3 and above, all patients were hospitalized and treated in various hospitals at Orlando Health. Blood samples were collected during patients’ stay in the hospital. The symptoms exhibited by the patient at the time of blood draw were documented. According to the clinical symptom severity, the patient was assigned with the Ordinal Scale from 3 to 7: 3—patients were hospitalized, but no oxygen therapy; 4—patients require oxygen by mask or nasal prongs; 5—patients require non-invasive ventilation or high flow mask; 6—patients require intubation and mechanical ventilation; 7—patients require ventilation and additional organ support such as vasopressors, RRT, and ECMO. We did not include samples from patients who died of COVID-19 in our study, which would be scale 8. In keeping with the Eight Category Ordinal Scale, patients with scale 1–2 were further grouped as asymptomatic/mild cohort (38 samples); patients with scale 3–4 are grouped as moderate cohort (54 samples); and patients with scale of 5 and above are group as severe cohort (61 samples).

### D2Dx immunity test of blood plasma samples

D2Dx immunity test kits (catalog D2Dx-hu-500, lot number hu08012020) were received from Nano Discovery Inc. (Orlando, Florida). Each kit contains the AuNP reagent and cuvettes for 500 tests. A handheld colorimeter reader device, CT-100 from Nano Discovery Inc. was used to read the test result. The specific composition and chemical structure of the AuNP reagent is proprietary information of Nano Discovery Inc. The AuNP reagent was manufactured, formulated, and calibrated using the CT-100 reader according to an internal quality control standard established by Nano Discovery Inc.

A 50 μL of the AuNP reagent solution was first placed into a cuvette using a micropipette. Then 10 μL of an undiluted blood plasma sample was added. After mixing the assay solution for 5 s using a mini-vortex mixer, the cuvette was placed in the cuvette holder in CT-100, and the result was read automatically in 30 s. The response of the test was reported directly as the absorbance change of the assay solution over a 30-s of reaction time.

### Kinetic interaction study of AuNP with IgG subclasses

The study of the interaction between the AuNP reagents and IgG subclasses from bovine, human and murine was conducted using the following materials: Bovine IgG1 (pep003, Bio-Rad, 1 mg/mL); bovine IgG2 (pep004, Bio-Rad, 1 mg/mL); human IgG1 (ab90283, Abcam, 3 mg/mL); human IgG2 (ab90284, Abcam, 2.2 mg/mL); human IgG3 (ab118462, Abcam, 2.2 mg/mL); human IgG4 (ab183266, Abcam, 1.5 mg/mL); mouse IgG1 (02-6100, Thermofisher, 1 mg/mL); mouse IgG2a (02-6200, Thermofisher, 1 mg/mL); mouse IgG2b (02-6300, Thermofisher, 1 mg/mL); mouse IgG3 (IMG5119A, Novus Biological, 0.5 mg/mL).

The kinetic study was conducted using a LaMotte model 3250 colorimeter. To an optical cuvette, 100 µL AuNP reagent from the D2Dx immunity test kit (D2Dx-hu-500) was added. Then 10 µL of the IgG subclass protein solution was added. Following mixing for 5 s using a mini vortex mixer, the cuvette was placed in the colorimeter, and the absorbance change of the assay solution was recorded every 30 s for a total reaction time of 3 min.

### SARS-CoV-2 specific antibody measurements

An enzyme-linked immunosorbent assay (ELISA) was used to detect SARS-CoV-2 specific IgG and IgM antibodies in plasma samples from patients (positive by PCR test with nasopharyngeal samples) and from donors with or without symptoms, who were tested positive or negative by PCR. Qualitative serology ELISA kits from Creative Diagnostics (Shirley, NY, USA) were used to measure anti-SARS-CoV-2 IgG (product # DEIASL019) and IgM (product # DEIASL020) levels in plasma samples. Briefly, 100 μL of negative and positive control were added to the positive and negative control wells without dilution. Ten microliters (10 μL) of plasma samples were added to the wells containing 100 µL of dilution buffer, mixed thoroughly, sealed with cover film, and incubated at 37 °C for 30 min. The plate was washed five times using 300 µL of 1 × diluted wash buffer. After completing all the assay steps according to the product instructions, the absorbance of the assay solutions at 450 nm was measured using a plate reader (Synergy H1 Hybrid Reader, BioTek, USA). An OD value of ≥ 0.50 for the positive control and ≤ 0.10 for the negative control sample were obtained, confirming the validity of the assay, per product instruction. The following cutoff values, as provided in the product manual, were used for results interpretation: < 0.3, negative; ≥ 0.3 to < 0.5, intermediate; ≥ 0.50, positive.

### Statistical analysis

Statistical differences of test results between different cohorts were analyzed using student t test, two-sample assuming unequal variances. P values < 0.05 were considered as significant difference. The numbers of asterisks indicate significance levels of P values, for example, the symbols of *, **, ***, and **** represent P values of ≤ 0.05, ≤ 0.01, ≤ 0.001, and ≤ 0.0001, respectively. If there is no significant difference (P > 0.05) between the groups, the results are presented as “ns”, namely, not significant.

Spearman’s rank-order correlation was used to analyze the correlation between the D2Dx immunity test scores of COVID-19 patients in the severe symptom cohort and the days from symptom onset to blood draw. The strength of the correlation was interpreted according to the scale suggest by Akoglu^47^: correlation coefficient 1—perfect; 0.7–0.9—strong positive correlation; 0.4–0.6—moderate positive correlation; 0.1–0.3—weak positive correlation; and 0—zero correlation. Both student t test and Spearman’s rank-order correlation was conducted using the data analysis function in Microsoft Office 2010 Excel software.
