# Author Correction: A 1-minute blood test detects decreased immune function and increased clinical risk in COVID-19 patients

**DOI:** 10.1038/s41598-021-04067-0

**Published:** 2021-12-21

**Authors:** Chirajyoti Deb, Allan N. Salinas, Tianyu Zheng, Aurea Middleton, Katelyn Kern, Daleen Penoyer, Rahul Borsadia, Charles Hunley, Bassam Abomoelak, Vijay Mehta, Laura Irastorza, Devendra I. Mehta, Qun Huo

**Affiliations:** 1 Translational Research and Specialty Diagnostic Laboratory, Arnold Palmer Hospital for Children, Orlando Health, 110 Bonnie Loch Court, Orlando, FL 32806 USA; 2 Nano Discovery Inc., 1060 Woodcock Road Suite 131, Orlando, FL 32803 USA; 3 Center for Nursing Research, Orlando Health, 1414 Kuhl Ave, Orlando, FL 32806 USA; 4 Center for Digestive Health and Nutrition, Arnold Palmer Hospital for Children, Orlando Health, 60 Gore St., Orlando, FL 32806 USA; 5 Internal Medicine Group, Orlando Health, 1414 Kuhl Ave, Orlando, FL 32806 USA; 6 Critical Care Medicine, Orlando Health, 1414 Kuhl Ave, Orlando, FL 32806 USA; 7 Department of Chemistry and NanoScience Technology Center, University of Central Florida, 12424 Research Parkway Suite 400, Orlando, FL 32826 USA

Correction to: *Scientific Reports* 10.1038/s41598-021-02863-2, published online 06 December 2021

The original version of this Article contained an error in the key for Figure 3, where the light green colour indicating “Severe” was incorrectly labelled as “D2Dx test score.”

The original Figure 3 and accompanying legend appear below.

**Figure 3:**
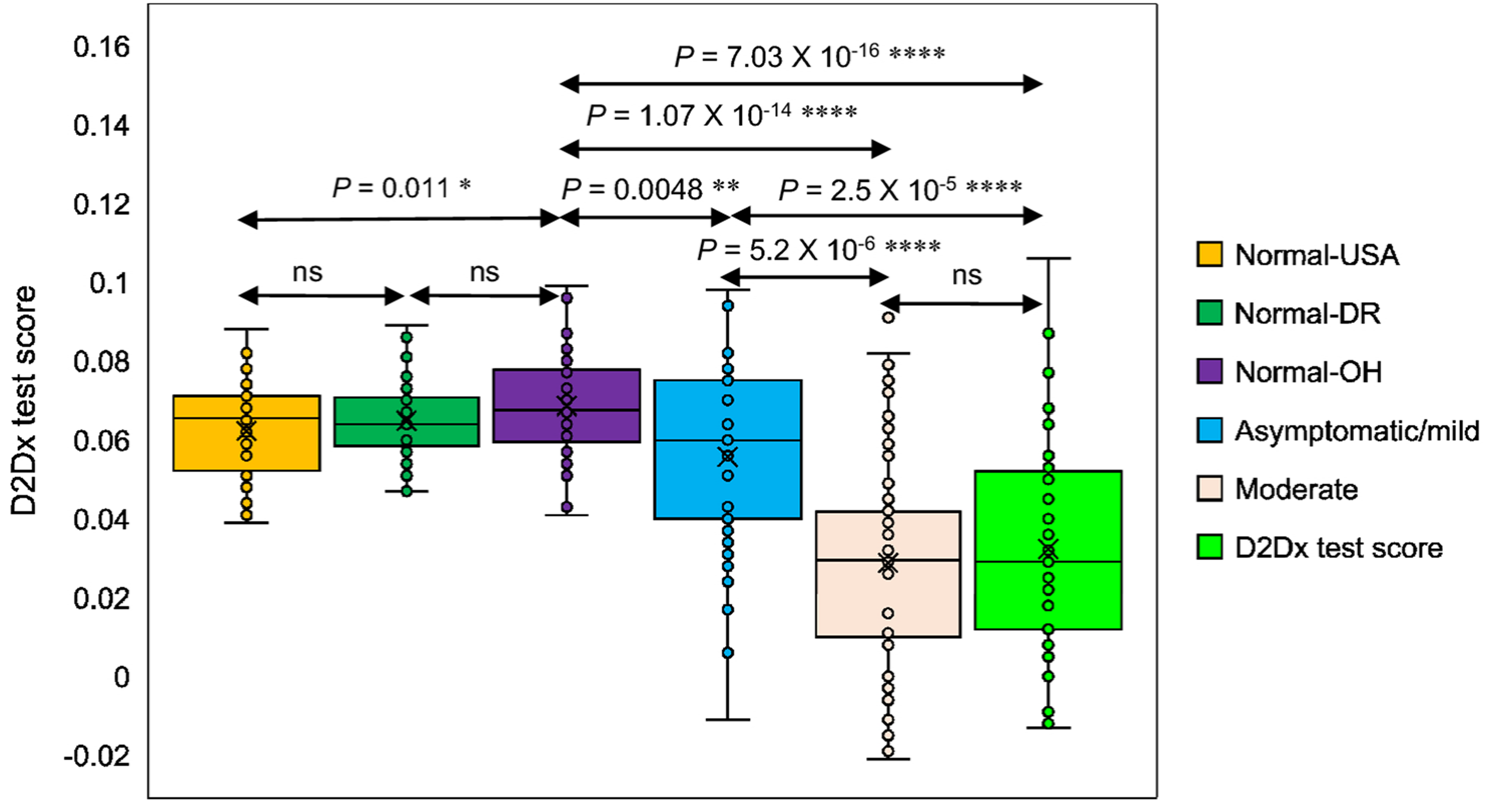
D2Dx immunity test response among various study cohorts. P values between different group pairs were calculated using student t test.

The original Article has been corrected.

